# Approaching Self-Bonded Medium Density Fiberboards Made by Mixing Steam Exploded *Arundo donax* L. and Wood Fibers: A Comparison with pMDI-Bonded Fiberboards on the Primary Properties of the Boards

**DOI:** 10.3390/ma16124343

**Published:** 2023-06-13

**Authors:** Federica Vitrone, Sascha Brinker, Diego Ramos, Francesc Ferrando, Joan Salvadó, Carsten Mai

**Affiliations:** 1 Department of Chemical Engineering, Rovira I Virgili University, Avinguda dels Països Catalans, 26, 43007 Tarragona, Spain; joan.salvado@urv.cat; 2 Department of Wood Biology and Wood Products, Georg-August-University of Göttingen, Büsgenweg 4, 37077 Gottingen, Germany; 3 Department of Mechanical Engineering, Rovira I Virgili University, Avinguda dels Països Catalans, 26, 43007 Tarragona, Spain

**Keywords:** medium density fiberboards, wood fibers, *Arundo donax* L., pMDI, steam explosion, self-bonding

## Abstract

This study presents an unexplored method to produce formaldehyde-free MDF. Steam exploded *Arundo donax* L. (STEX-AD) and untreated wood fibers (WF) were mixed at different mixing rates (0/100, 50/50, and 100/0, respectively) and two series of boards were manufactured, with 4 wt% of pMDI, based on dry fibers, and self-bonded. The mechanical and physical performance of the boards was analyzed as a function of the adhesive content and the density. The mechanical performance and dimensional stability were determined by following European standards. The material formulation and the density of the boards had a significant effect on both mechanical and physical properties. The boards made solely of STEX-AD were comparable to those made with pMDI, while the panels made of WF without adhesive were those that performed the worst. The STEX-AD showed the ability to reduce the TS for both pMDI-bonded and self-bonded boards, although leading to a high WA and a higher short-term absorption for the latter. The results presented show the feasibility of using STEX-AD in the manufacturing of self-bonded MDF and the improvement of dimensional stability. Nonetheless, further studies are needed especially to address the enhancement of the internal bond (IB).

## 1. Introduction

Formaldehyde emissions are still a major concern when producing wood-based panels (WBP) [1]. Indeed, the use of formaldehyde-based adhesives has been widely associated with the release of formaldehyde, which causes a serious hazard to human and environmental health (ordinance EU 605/2014) [2]. The most widely applied solution in industry is to use formaldehyde scavengers, which allow the formulation of adhesives that do not exceed the level of emissions permitted by the regulations. Urea is the most common scavenger employed in industry, but other amine compounds were studied for the purpose and applied in WBP [3,4,5,6]. On this issue, both academia and industry devoted a special effort towards sustainable WBP, which are less hazardous, but qualitatively comparable to current industrial products [7]. As regards the literature review of the last 15 years, this topic has been widely studied finding several solutions, including (i) the use of synthetic formaldehyde-free adhesives, bio-based adhesives (e.g., proteins, starches, tannins, and lignin) or a combination of both of them to partially or totally replace fossil resins [7,8]; (ii) taking advantage of the intrinsic self-bonding properties of lignocellulosic materials to manufacture totally binder-free boards [7].

The bonding of solid wood and wood particles is a key factor in achieving the standards imposed by the regulations [9]. From this viewpoint, the total replacement of formaldehyde-based adhesives is still challenging. This is due to the low reactivity of bio-adhesives, which consequently leads to an increase in production time and costs, as well as lower mechanical properties and dimensional stability [1]. From an industrial perspective, the low reactivity of bio-adhesives does not make them immediately usable, unless by modification or the use in combination with a synthetic crosslinker, aiming to promote the formation of intermolecular bonds between polymer chains [9,10]. For instance, many studies have been conducted to replace formaldehyde-based adhesives with alternatives such as glyoxal-based adhesives, creating a new generation of environmentally friendly adhesives [11], also employed in combination with natural compounds, such as tannin [12], lignin [13], and soybean [14].

Among the synthetic adhesives, polymeric methyl diphenyl diisocyanate (pMDI) gained increasing popularity as it shows a higher affinity to wood and non-wood materials than formaldehyde-based resins [15,16]. pMDI has the advantage of reacting with the naturally occurring moisture inside the wood and lignocellulosic materials to form polyurea networks [17,18], although the reactions that contribute to bond formation have not yet been fully elucidated. Moreover, other benefits associated with pMDI are the fast curing rate, the good moisture tolerance, and the excellent dry and wet bonding strength [8,18], all of which made it an outstanding formaldehyde-free alternative to traditional binders. On the other hand, certain drawbacks need to be addressed. Firstly, the potential health hazard and carcinogenicity is still under study [8,19]. Furthermore, its cost is higher than conventional formaldehyde-based adhesives, thus leading to more expensive final products [20,21]. In this framework, research paid attention to the formulation of new adhesives obtained by combining pMDI and bio-adhesives for both reducing the amount of pMDI, thereby the cost of board manufacturing, and increasing the reactivity and water resistance of natural binders.

Hidayat et al. [22], studied the feasibility of natural rubber latex (NRL) as an adhesive by adding 5% of pMDI to the adhesive formulation. They showed the suitability of producing particleboards with 20% NRL/pMDI related to dry particle mass, although the enhancement of mechanical and physical properties needed further studies and they attributed the poor properties to the low percentage of pMDI in the adhesive formulation. On the other hand, Hemmilä et al. [10], compared the effect of two different crosslinkers, i.e., pMDI and furfuryl alcohol (FA), in combination with ammonium lignosulfonate (ALS). They used a ratio of 6% for both pMDI and FA to ALS for a total amount of 12% of adhesive (wt% to dry particles), thus aiming to reduce it to 8%. Hence, they showed the superiority of pMDI as a crosslinker for ALS in achieving a higher IB, with further improvements by adding tannin to the adhesive formulation. Moreover, Asafu-Adjaye et al. [20], used the pMDI-based binder in the amount of 2 wt% and 4 wt% to dry particles, partially substituting it with 12% and 15% of soy flour. They compared the results obtained with the different amounts of soy flour plus pMDI, with pure pMDI bonded boards in the same amount. The mixing of soy flour and pMDI resulted in the improvement of all the properties for both strand boards, MDF, and particleboards. Ostendorf et al. [23], used the same amounts of pMDI and showed the improvement in IB and a sufficient performance against moisture by doubling its amount in boards made of thermomechanical pulp fibers of spruce and silver fir, kraft lignin (i.e., technical lignin) as adhesive, and pMDI as crosslinker.

Overall, technical lignin itself is considered one of the most promising alternatives to formaldehyde-based binders [24], and it has already been studied for the formulation of formaldehyde-free binders for board production [25]. Recent review papers [26,27] explored the lignin application in wood adhesives and wood modification, drawing attention to the gap between the potential of this abundant natural polymer and its actual use at the pilot scale. The increasing interest in lignin in the field of adhesives is due to its polyphenolic structure, which can partially substitute the phenol in phenol-formaldehyde resin [27,28,29]. Lignin is abundant, promising, and one of the main components of lignocellulosic materials, together with cellulose and hemicelluloses [26].

Research has also been focusing on the self-bonding properties of wood and non-wood fibers, due to the lignin and sugars of the lignocellulosic structure itself [7,30,31]. In the natural form of wood, as well as other lignocellulosic materials, lignin already acts as a binder, but it is incorporated in a polymer chain with cellulose and hemicellulose, which together give the wood its natural resistance to loads and moisture, with some differences depending on the resource considered [32]. Consequently, the pretreatment of the lignocellulosic material is necessary to decompose the chain, making the self-bonding attainable, and to reduce the number of impurities aiming to improve final properties [30,33]. Many pretreatments have been the subjects of extensive research, such as hydrothermal, biological, and enzymatic pre-treatments [34].

Among the hydrothermal pre-treatments, steam explosion (STEX) has been used for years, as the Masonite process, in the production of fiberboards [35]. Hence, STEX already showed its applicability in this field, especially in the production of hardboards through wet processes, with many examples available in the literature, and applications with different materials such as residual softwood [36], grey alder wood [37], banana bunch [38], bamboo chips [35,39], hemp shives and wheat straw [19,40], rice straw [41], and *Arundo donax* L. [42,43,44,45].

The success of STEX when producing self-bonded boards is due to the hydrothermal reactions provided during the hot steam supply and the sudden decompression. These two steps are able to break the biomass linkage, partially hydrolyze the hemicellulose, and change the lignin structure by depolymerization/repolymerization reactions [33], thus enhancing the self-bonding mechanism by physical consolidation and chemical activation [46]. Although the STEX prior to the manufacture of WBP has already proven to be industrially viable, as well as environmentally friendly [47], its application is limited mostly to thin hardboards made by a wet process, and the results presented in the aforementioned studies might not compete in the market of WBP.

To the best of our knowledge, there are few studies in literature using STEX as a pre-treatment in the manufacturing of MDF, i.e., by a dry process. On this basis, the present work aims to explore the possibility of using STEX in the production of MDF, comparing the results obtained with pMDI-bonded boards. As a first approach, the mechanical and physical properties of MDF made by mixing STEX-AD and conventional WF in different percentages were studied. Indeed, in several studies [42,44,45,48], high-quality panels were obtained by using STEX-AD. Thus, the purpose of the present work is to identify any enhancements triggered by treating the material, and whether the lack of the adhesive can be compensated when mixing pretreated and untreated materials. Very interesting initial results are presented that lay the foundation for future improvements.

## 2. Materials and Methods

### 2.1. Preparation of the Materials

WF was kindly provided by Gutex^®^ Holzfaserplattenwerk H. Henselmann GmbH and Co. KG (Waldshut-Tiengen, Germany). Gutex thermofibre^®^ is composed of refined pine (*Pinus sylvestris* L.) and spruce (*Picea abies* (L.) H. Karst.) at a mixing ratio of 95% to 5%.

STEX-AD (55.4 ± 0.9% cellulose, 29.0 ± 0.9% lignin, 5.8 ± 0.8% hemicelluloses) were produced at the University Rovira i Virgili (Tarragona, Spain). The untreated reeds were provided by Cañizos Albatera SL (Mos del Bou, Spain) and were collected from Ribarroja de Turia, Valencia (Spain). Untreated AD is generally composed of 29.2–43.1% of cellulose, 19.2–24.3% lignin, and 14.5–32.0% of hemicellulose, depending mainly on age [45,49,50,51]. The STEX process performed has already been described in other papers [44,45,48] and the process parameters used in this study were the pre-treatment temperature (Tr) of 200 °C and the time (tr) of 9.5 min.

The pMDI I-Bond WFI 4370 was used as a crosslinker and co-binder for the reference panels and it was obtained from Huntsman Corporation (Everberg, Belgium).

The WF and the STEX-AD were shredded in the Electra hammer mill (Poudenas, France) with a 10 mesh, as it has been shown in previous studies [52] that milled material leads to improvements in IB and the moisture before mixing and pressing was approximately 7% and 8%, respectively.

### 2.2. Medium Density Fiberboards Manufacturing

Six different kinds of MDF were produced by using solely wood fibers (WF) and STEX-AD and their mixture with and without pMDI, i.e., 4 wt% related to the dry fiber mass (Table 1).

In all cases, the process encompassed blending the fibers in a rotating drum blender for 5 min at a speed of 30 rounds/min, spraying pMDI for the adhesive bonded samples, and water to adjust the moisture content from the initial 8% to 11% (necessary to ensure the formation of polyurea adhesive from pMDI) and homogenous mixing of the fibers both with and without the binder.

After blending, the fibers were pre-pressed into a mat and then hot pressed (Joos LAP 40, Pfalzgrafenweiler, Germany) at 205 °C for 15 s mm^−1^ with a pressure of 5 N mm^−2^, to form a board of 250 × 250 × 11 mm^3^. Metal bars were used as stops and the target density was set at 780 kg m^−3^.

**Figure 1:**
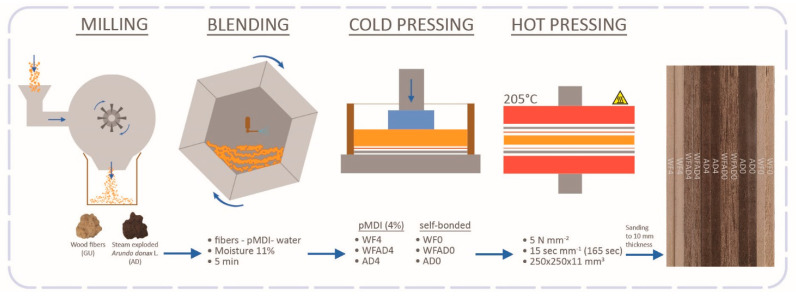
Production scheme of medium–density fiberboards (Adapted from [53]).

The boards obtained were cut into specimens that were sanded to equalize the thickness to 10.5 ± 0.5 mm. The samples were then conditioned at 20 °C and 65% relative humidity to be tested after conditioning. The entire manufacturing process is illustrated in Figure 1.

### 2.3. Physico-Mechanical Characterization

The European (EN) standards were followed to characterize the specimens. The density was calculated according to EN 323:1993 [54] for five specimens per panel. Vertical density profiles (VDP) were obtained using an X-ray densitometer (DAX, Fagus-Grecon GmbH and Co. KG, Alfeld, Germany) by 50 × 50 × 10.5 ± 0.5 mm^3^, and the density was measured at intervals of 0.01 mm along with the thickness. Mechanical properties were calculated according to EN 310:1994 [55], for modulus of elasticity (MOE) and modulus of rupture (MOR), and EN 319:1994 [56] was used as a reference for IB. The three-point bending test was performed on specimens measuring 240 × 50 × 10.5 ± 0.5 mm^3^ (four repetitions for each sample), while eight specimens for each sample of 50 × 50 × 10.5 ± 0.5 mm^3^ were glued on metallic braces to evaluate the IB. MOE, MOR, and IB were obtained by using the universal testing machine Zwick-Roell Z010 (Zwic-Roell, Ulm, Germany). Thickness swelling (TS) after 2 h, 24 h, and 48 h water immersion were determined according to EN 317:1994 [57] using eight specimens for each sample of 50 × 50 × 10.5 ± 0.5 mm^3^.

EN 622-5:2010 [58] was used as a reference for the minimum and maximum values required for MDF, intended for use in dry (Type MDF) and wet (Type MDF.H) conditions.

### 2.4. Statistical Analysis

One-way Analysis of Variance (ANOVA) was performed for physical, bending, and internal bonding properties of the produced MDF, using Rstudio (version 3.6.3), at a level of significance of α = 0.05 considering the material as the six-level factor (named: WF4, WF0, WFAD4, WFAD0, AD4, and AD0). Subsequently, Tukey’s Honestly Significant Difference (HSD) was chosen as a multiple comparison posthoc test to evaluate the significant difference between the groups. On the one hand, the means of the values among the specimens with the same fiber formulation were compared (i.e., WF4-WF0, WFAD4-WFAD0, and AD4-AD0). On the other hand, the means of the values of the specimens within the two groups with and without the adhesives were also compared (i.e., WF0-WFAD0-AD0, and WF4-WFAD4-AD4).

## 3. Results and Discussion

### 3.1. Density and Mechanical Properties

**Figure 2:**
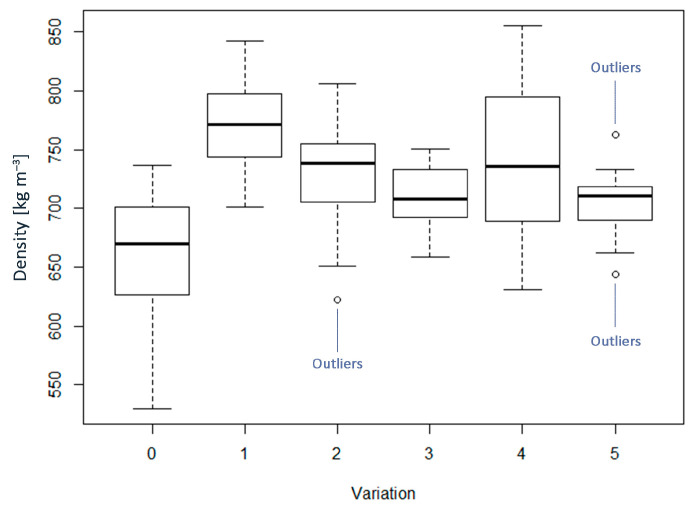
Boxplots of density for each of the variations considered. In the x–axis: 0 = WF0; 1 = WF4; 2 = WFAD0; 3 = WFAD4; 4 = AD0; 5 = AD4.

The average density of the samples ranged from 665 to 769 kg m^−3^, being in the range of conventional MDF (Figure 2). The lower density of some variants, especially WF0, was due to the thickness spring-back after pressing. This can be explained by the low adhesion of the fibers to each other, which occurs for self-bonded panels made from untreated material.

The highest density was obtained for the sample WF4, followed by AD0, WFAD0, WFAD4, AD4, and WF0 (Figure 2). The AD0 sample was the one within self-bonded boards with the density closest to the maximum obtained for WF4. This can be explained by the compressibility of AD after STEX. Indeed, various Tr have been studied for the pretreatment of AD [44]. It has been shown that starting from a certain Tr, a sufficiently defibrillated material is obtained, which allows for the production of panels with a higher density compared to those made with material exploded at lower Tr. This facilitates achieving the target thickness more easily. These panels, due to the chemical and morphological changes of the material, exhibit improved mechanical behavior. Specifically, lignocellulosic material treated by STEX results in a defibrillated pulp for which the fibers are thin and long [39,59], thus enhancing the compactness of the boards. Domínguez-Robles et al. also related the increase in density to the decrease in the length and width of the fibers, and to the increase of fine elements as a consequence of the pretreatment, thus observing an improvement in mechanical strength with respect to boards made of untreated fibers [60].

The ANOVA showed that the material formulation had a major influence on density (Table 2). By the multiple comparisons, we found that the density values that stand out from the others are those of WF0 and WF4 for being the lowest and highest, respectively. Therefore, the density of WF0 is significantly different from WFAD0, AD0, and WF4, and the density of WF4 is significantly different from WFAD4, AD4, and WF0. On the other hand, the statistically equal densities are WFAD0-AD0, WFAD4-AD4, WFAD0-WFAD4, and AD0-AD4 (Table 2). Thus, comparing the same fiber formulation with and without adhesive, the difference in density was significant only when using only WF, whereas for specimens containing AD, there was no difference in density. The analysis of the significant differences with respect to density leads to a better understanding of the differences in mechanical properties since density is directly related to mechanical performance. Indeed, in the case of self-bonded samples, the density increased by adding exploded AD, while in the case of pMDI-bonded samples, exactly the reverse occurred, and the density decreased as exploded AD was added. This could indicate low compatibility of the exploded material with this type of adhesive, i.e., pMDI, consequently affecting all other mechanical properties.

**Figure 3:**
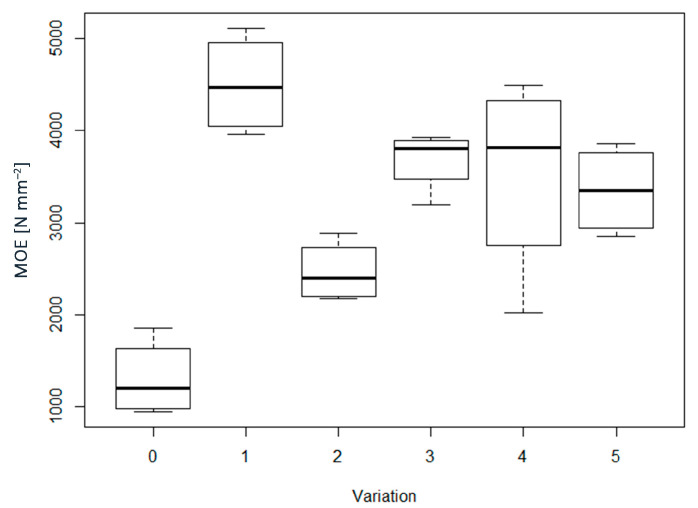
Boxplots of modulus of elasticity (MOE) for each of the variations considered. In the x–axis: 0 = WF0; 1 = WF4; 2 = WFAD0; 3 = WFAD4; 4 = AD0; 5 = AD4.

Figure 3 shows the boxplots of Modulus of elasticity (MOE). The highest MOE was obtained for the sample WF4, which reached an average value of 4497 N mm^−2^. The density, statistical analysis showed a significant *p*-value (Table 2). Based on these results, boards made with pMDI showed the highest MOE values except for AD0, which had an MOE in the same range as WFAD4 and AD4, as also shown by the multiple comparisons (Table 2). The increase in MOE can be related to the increase in density, as well as to other mechanical properties, which are connected with the bonding quality and the adhesion between fibers [15,18,22,29,61,62]. This is especially noticeable when comparing self-bonded and pMDI-bonded boards, where MOE showed the same significant differences in density between the groups considered: where there was a difference in density, there was a difference also in MOE values, which increased for higher density. However, for the pMDI-bonded boards, some differences were found although there was a significant difference in density between WF4 and the other two groups (i.e., WFAD4, and AD4), the samples containing AD did not show any difference in MOE values with WF4. With regard to self-bonded boards, the only deviation with respect to the dependence of MOE on density occurred for the WF0 and WFAD0 groups, whereby there was no significant difference in MOE values while there was for density. Referring to the standard EN 622-5:2010, all the samples made with pMDI met the requirements for structural use both in dry and wet conditions, while for the self-bonded samples only AD0 reached the minimum value required (i.e., 2700 N mm^−2^), although sample AD4 was very close to it.

**Figure 4:**
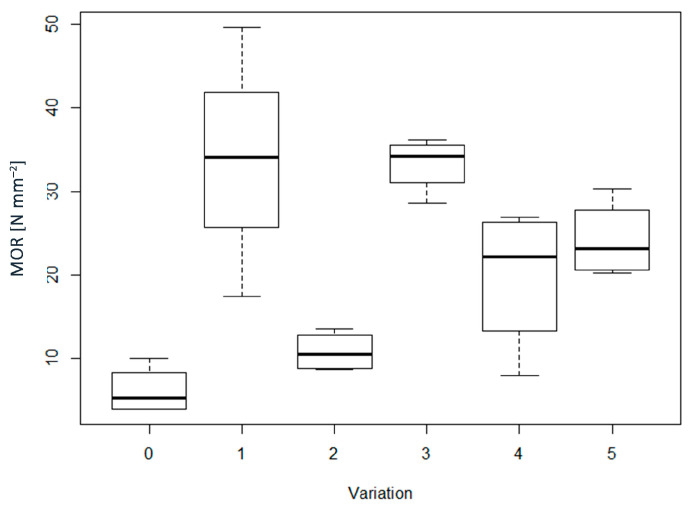
Boxplots of modulus of rupture (MOR) for each of the variations considered. In the x–axis: 0 = WF0; 1 = WF4; 2 = WFAD0; 3 = WFAD4; 4 = AD0; 5 = AD4.

For Modulus of Rupture (MOR) (Figure 4) the relationship with the presence or absence of adhesive was clearer. Additionally, here the higher average value was found for sample WF4 (i.e., 33.7 N mm^−2^), and the variation had a significant effect on the MOR results, although mostly because of the adhesive (Table 2). Indeed, there were no significant differences between the WF0, WFAD0, and AD0 groups, as well as between the WF4, WFAD4, and AD4 groups, although there were differences in density for some of these groups (Table 2). Whilst for MOE there was the possibility that the percentage of AD had an influence on the values obtained, for MOR it was clear that it depends prevalently on the adhesive in the case where wood fibers were used, while for the AD4 and AD0 groups, not even the presence of the adhesive resulted in differences in the MOR value. Additionally, referring to the standard EN 622-5:2010, only pMDI-bonded samples achieved the required level for MOR values (i.e., 22 N mm^−2^ for type MDF, and 27 N mm^−2^ for type MDF.H).

**Figure 5:**
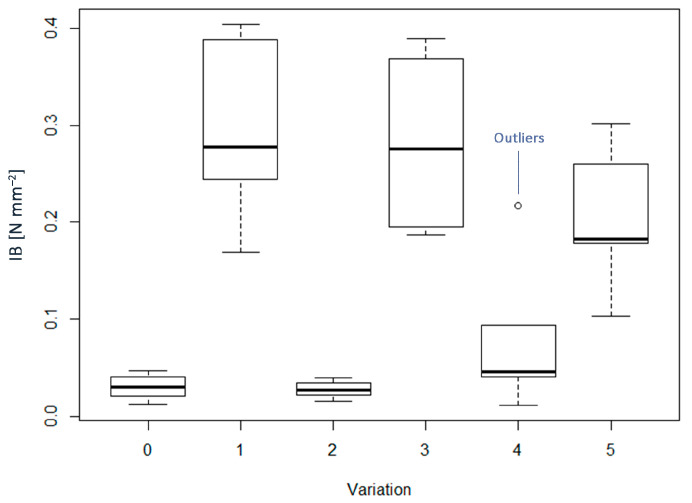
Boxplots of internal bond (IB) for each of the variations considered. In the x–axis: 0 = WF0; 1 = WF4; 2 = WFAD0; 3 = WFAD4; 4 = AD0; 5 = AD4.

The Internal Bond (IB) was very low in each case and none of the samples came to the value set by the standard (Figure 5), as the minimum for general use is 0.65 N mm^−2^. In addition, a large dispersion in values was found, particularly for pMDI-bonded samples, possibly due to the low homogeneity of the boards and the alleged low uniform distribution of the resin. Therefore, for statistical analysis, IB results that deviated significantly from the average were excluded from the collected data, resulting in six to eight values per sample. The dispersion in IB values is much higher for pMDI-bonded samples (Figure 5), especially for WF4 and WFAD4. On this subject, Medved et al. [63] conducted extensive research to analyze the impact of resin content on the adhesive’s surface coverage. They investigated the relationship between coverage and fiber shape, size, and adhesive content by introducing a fluorescent dye into the UF adhesive. By examining the fibers under a fluorescent light source, they observed the highest surface coverage on larger fibers with a higher resin content, which amounted to 12.5% to dry particles mass. However, the unexpectedly low values obtained also for pMDI-bonded boards were in line with some works found in the literature [20]. Overall, the samples showed the same tendency as the MOR, appearing highly dependent on the presence of the adhesive. Indeed, when comparing WF0, WFAD0, and AD0, no significant differences were found, as well as between groups WF4, WFAD4, and AD4. On the other hand, when comparing the same material formulation with and without the adhesive there was a strong significant difference between WF0 and WF4, and between WFAD0 and WFAD4. Conversely, the significant difference between groups AD0 and AD4 was weakly significant, with a *p*-value of 0.03. Certainly, further studies are needed to improve this property. Comparing these results with other studies found in the literature [15,64,65], an approach may be using longer pressing times. Indeed, the penetration of the heat of the press into the core layers is essential for proper bonding and an increased pressure time makes the heat penetrate along the thickness. Other studies also showed the improvement in IB by adding ammonium lignosulphonate or kraft lignin [10,23,66], thus obtaining IB values above the threshold.

### 3.2. Dimensional Stability

**Figure 6:**
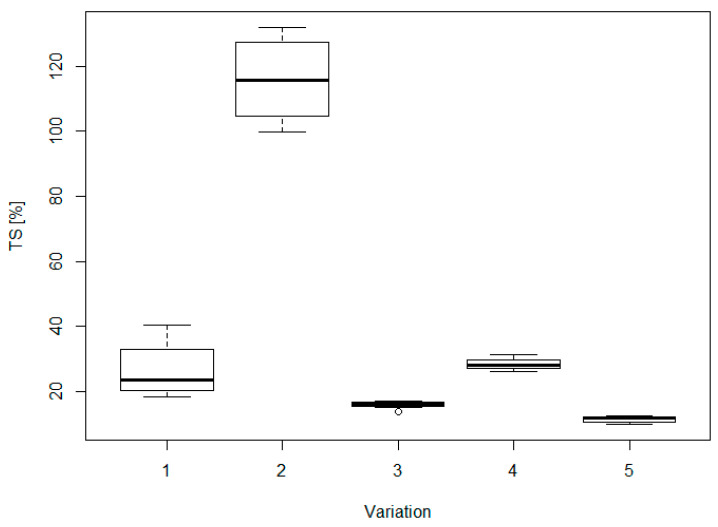
Boxplots of thickness swelling (TS) for each of the variations considered. In the x–axis: 1 = WF4; 2 = WFAD0; 3 = WFAD4; 4 = AD0; 5 = AD4.

Thickness swelling (TS) and water absorption (WA) are critical parameters correlated with dimensional stability, for which it is more difficult to achieve the requirements of the standards when using alternative materials and adhesives [15,67,68,69]. Indeed, only version AD4 fulfilled the limit of TS at 24 h for general and structural use in dry conditions, set at 15% (Figure 6), while for sample WF0 it was not even possible to measure the TS, since the specimens became completely detached after a few minutes of immersion.

Previous studies have proved [39,41] that the STEX prior to the manufacturing of fiberboards has the great advantage of improving dimensional stability. The results obtained showed the improvement of TS and WA values in the samples made with 100% and 50% of steam-exploded AD. Moreover, AD as a raw material has a high percentage of lignin (19–24% lignin, 29–43% cellulose, 14–32% hemicellulose). In this way, the combination of AD and STEX is particularly beneficial for TS, since lignin is more hydrophobic than polysaccharides [70].

More specifically, with the STEX pretreatment, the primary components of the lignocellulosic material are separated, the highly hydrophilic hemicelluloses are mostly hydrolyzed and washed after the treatment, and the lignin is more evenly distributed over the cellulose structure, creating a hydrophobic layer [41].

**Figure 7:**
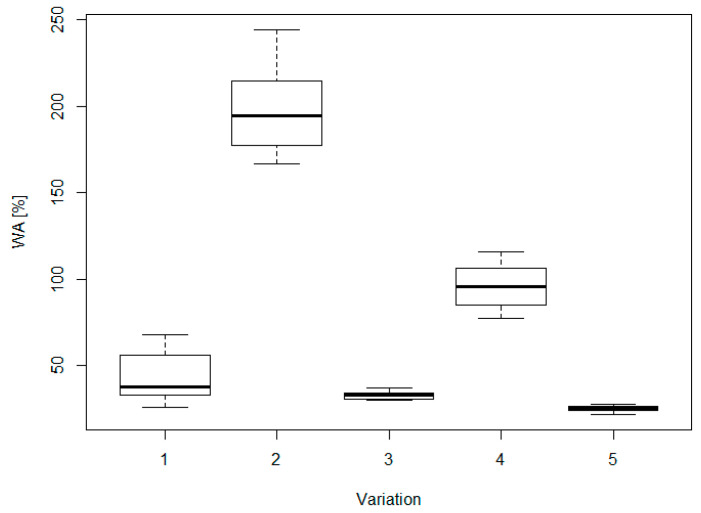
Boxplots of water absorption (WA) for each of the variations considered. In the x–axis: 1 = WF4; 2 = WFAD0; 3 = WFAD4; 4 = AD0; 5 = AD4.

For TS, as well as for WA, the lowest values at 24 h were obtained for the sample AD4 and WFAD4, which were 11% and 16% for TS, and 25% and 33% for WA, respectively. Both TS and WA showed a clear decreasing trend when comparing within the group with pMDI, i.e., WF4, WFAD4, and AD4, as well as the group without pMDI, i.e., WFAD0, AD0 (Figure 6 and Figure 7). Although no numerical results are available for sample WF0, the fact that it detached indicates in any case that the addition of AD resulted in an improvement in dimensional stability, although not sufficient to achieve the standard.

The ANOVA results (Table 3) show a high influence of the variation on the properties considered. Through the multiple comparisons (Table 3) the differences between the specific groups can be found, and thus whether the significant difference is due to fiber formulation or the presence of the adhesive.

As for TS, there was a significant difference between all groups, except AD4 and WFAD4. There was a clear improvement in swelling thanks to both the addition of AD and pMDI. Indeed, while in the comparison of samples with pMDI the differences were minor (only between WF4 and WFAD4 and with a *p*-value of 0.03), the difference between WFAD0 and AD0 was much more pronounced, where AD0 had an average value of TS at 24 h comparable to WF4.

**Figure 8:**
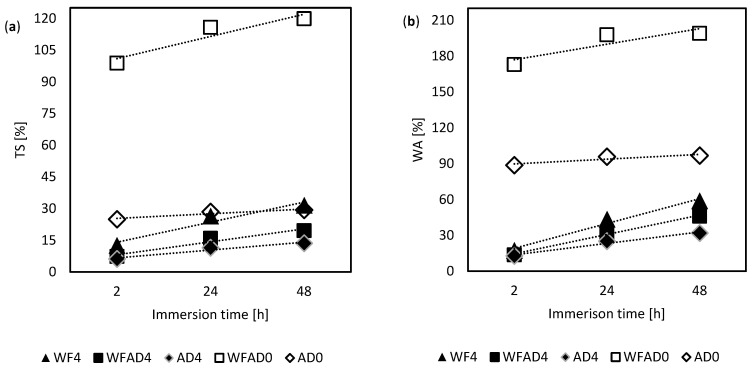
Thickness swelling (**a**) and water absorption (**b**) increasing in 2 h, 24 h, and 48 h of water immersion, for each of the variations considered.

WA results were similar to TS, but there was no difference between the groups with pMDI, i.e., WF4, WFAD4, and AD4, while the TS at 24 h of AD0 was about half of WFAD0, and comparable to WF4. Considering the AD0 sample, the anomalous finding was the mismatch between TS and WA: AD0 had a relatively high WA but a relatively low TS, which is not found in the pMDI-bonded samples. The main difference between self-bonded and pMDI-bonded samples was in the initial absorption and swelling (Figure 8): pMDI-bonded boards absorbed water at a slower pace than self-bonded ones, for which the WA and TS at 2 h and at 48 h were almost the same. Although WF4 and AD0 samples achieved about the same value of TS at 24 h, the AD0 sample showed a higher short-term absorption, suggesting that AD0 reached the equilibrium status of saturation in a few hours (i.e., TS for AD0 is 25%, 28%, and 29%, at 2 h, 24 h, and 48 h, respectively). Indeed, AD0 had already reached its maximum value of TS after 2 h. Thus, it remained approximately the same, while WF4 swelling kept increasing to 32% at 48 h.

### 3.3. Vertical Density Profiles

Vertical Density profiles (VDP) show density distribution along the thickness, and they were recorded to find out a correspondence between the densities and the mechanical parameters, especially MOR and IB. Indeed, the IB depends on the bonding of the core layer [1,62,71], the MOR and the MOE are mostly influenced by the strength of the face layers [72], although the MOE depends also on the material itself. A typical MDF density profile presents two peaks at the face layers where the density is the highest, while in the core layer, the density drops to its lowest value [1,62]. This has been associated in other studies with the higher compaction in external regions due to the direct contact with the hot plates, as well as with the smaller fiber size and higher resin content [64].

**Figure 9:**
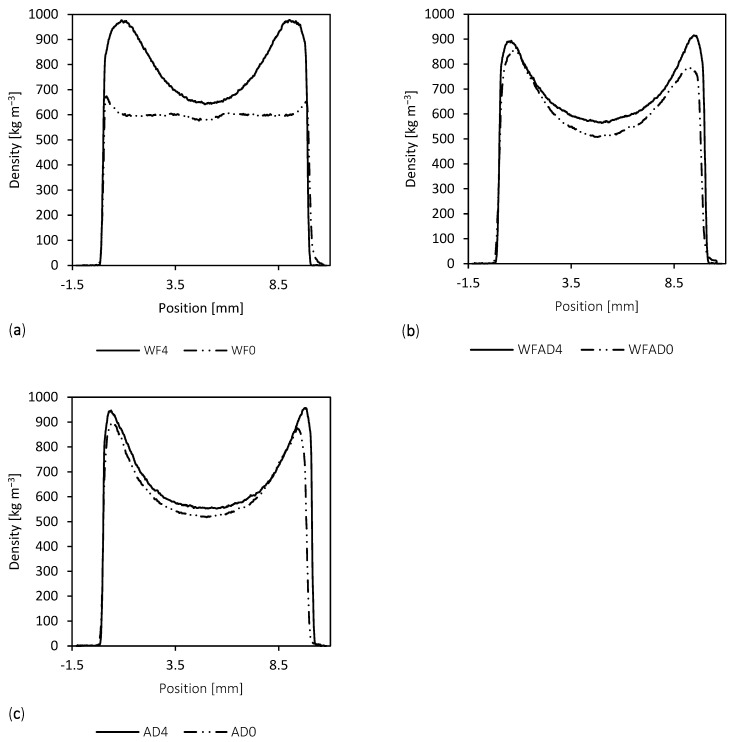
Vertical density profiles of samples WF4–WF0 (**a**), WFAD4–WFAD0 (**b**), and AD4–AD0 (**c**).

Profiles were compared either by pairing the same fiber formulation with and without the adhesive, and by grouping specimens with the adhesive and those without (Figure 9). All samples showed the typical U-shape of MDF, except the WF0, for which the density was about the same along the thickness (Table 4). This can partly explain the higher MOR of self-bonded boards made with a higher percentage of steam-exploded AD and shows the steam-exploded AD gluing ability, activated by the heating during hot pressing [73]. The sanding may have affected the shape of the VDP, especially in the WF0 sample. This may have altered the mechanical properties of WF0, which, after hot pressing, suffered a spring back of the thickness greater than the other samples. Indeed, the sanding probably removed the outermost layers, which are normally the denser ones [62], and also the ones related to MOE and MOR properties. However, sanding is a step that is normally used at the industrial level, and it is necessary to equalize the surfaces and thicknesses of the panels that will be sold on the market. Thus, considering that the temperature and pressure time were the same for all samples, these results may be due to the steam exploded AD which limited the spring back after pressing and thus enhanced a better bonding. There may be several reasons for this, among them the increased presence of fine particles in the exploded material, and the agglomerating effect of lignin distributed on the fiber surface due to the STEX.

The density of self-bonded boards was for each sample, and in every position, lower than pMDI-bonded boards, a result that can be easily connected with mechanical performance: both MOR and IB were higher for pMDI-bonded boards, as pMDI led to a better cohesion of the fibers.

Comparing the different formulations with and without adhesive, there was also a match in mechanical properties: the specimens that showed a better profile were WF4 and AD0, which were those that, within their own group, pMDI-bonded and self-bonded, respectively, showed the best mechanical performance.

## 4. Conclusions

The result presented represent a first exploration both in term of the material used and the method. The performance obtained did not reach the expected standard, but it can be considered as a starting point to identify the critical points for further optimization.

Based on the results, the combination of exploded AD with pMDI does not produce the same effect as the combination of WF with pMDI: WF is much more compatible with this type of adhesive than AD. Indeed, when comparing WF0 and WF4 the differences are much higher in both mechanical properties and dimensional stability. On the other hand, for AD0 and AD4 there is only a small difference in IB, for mechanical properties, while the differences are high for dimensional stability, as the synthetic adhesive has better hydrophobic properties.

The density and the adhesive content showed a major influence on the mechanical properties and dimensional stability. The mixing of WF and AD resulted in the increase in density and the enhancement of mechanical properties for self-bonded boards, compared to those solely made from WF. Nevertheless, the mechanical properties of adhesive-free MDF containing AD did not reach the performance level of pMDI-bonded panels. As for the dimensional stability, self-bonded MDF made solely of AD (i.e., AD0) can attain TS values comparable to WF4, although the WA of the former is significantly higher. Moreover, the panels made by AD without the adhesives showed a higher short-term absorption than all the pMDI-bonded boards. Although this latter is not a good result for MDF, it is an interesting result that may lead to applications in other fields. The AD can improve the swelling for both pMDI-bonded and binder-free boards and the sample AD4 was the only one achieving the standard for MDF in dry conditions, although none of the samples reached the value set for wet conditions. The IB was low in any case and the results obtained did not achieve the thresholds of the regulations. Nonetheless, an improvement was observed for pMDI-bonded panels.

Further studies need to be developed aiming toward the enhancement of both mechanical properties and dimensional stability. It is necessary to focus on WA and IB in order to find new approaches and new applications, aspiring to the exploitation and valorization of the potential of this material.

## Figures and Tables

**Table 1:** Mixing design of the medium density fiberboards.

Variations	WF	AD	pMDI
	wt%	wt%	wt%
WF0	100	0	0
WF4	96	0	4
WFAD0	50	50	0
WFAD4	48	48	4
AD0	0	100	0
AD4	0	96	4

**Table 2:** One-way ANOVA and HSD *p*-values for density and mechanical results (MOE, MOR, and IB) considering the variation as a six level factor.

Multiple Comparison Pairs	ρ	MOE	MOR	IB
	[kg m^−3^]	[N mm^−2^]	[N mm^−2^]	[N mm^−2^]
	ANOVA *p*-Value	ANOVA *p*-Value	ANOVA *p*-Value	ANOVA *p*-Value
	1.56 × 10^−9^	1.18 × 10^−5^	0.0002	2.80 × 10^−8^
WF4-WF0	<0.0001	<0.0001	0.0013	<0.0001
WFAD4-WFAD0	0.7153	0.0876	0.0044	<0.0001
AD4-AD0	0.1397	0.9975	0.9525	0.0334
WFAD0-WF0	0.0002	0.1119	0.9393	0.9999
AD0-WF0	<0.0001	0.0006	0.1325	0.8362
AD0-WFAD0	0.9897	0.1622	0.5107	0.7568
WFAD4-WF4	0.0011	0.4070	0.9999	0.9994
AD4-WF4	0.0002	0.1172	0.5347	0.2782
AD4-WFAD4	0.9977	0.9671	0.4998	0.5272

**Table 3:** One-way ANOVA and HSD *p*-values for thickness swelling (TS) and water absorption (WA) considering the variation as a six level factor.

Multiple Comparison Pairs	TS	WA
	[%]	[%]
	ANOVA *p*-Value	ANOVA *p*-Value
	<2 × 10^−16^	<2 × 10^−16^
WFAD4-WFAD0	<0.0001	<0.0001
AD4-AD0	0.0002	<0.0001
AD0-WFAD0	<0.0001	<0.0001
WFAD4-WF4	0.0307	0.61
AD4-WF4	0.0009	0.1284
AD4-WFAD4	0.6967	0.851

**Table 4:** Peak densities, core density, and average density for each of the variants considered.

Series Name	Peak Density (Left)	Peak Density (Right)	Core Density	Average Density
	kg m^−3^	kg m^−3^	kg m^−3^	kg m^−3^
WF0	666	652	582	662
WF4	975	971	648	806
WFAD0	846	784	510	739
WFAD4	892	909	567	705
AD0	887	862	520	682
AD4	945	950	567	710

## Data Availability

The data presented in this study are available on request from the corresponding authors.
